# Linking childhood emotional maltreatment to social media addiction: a network perspective on executive function and physical activity

**DOI:** 10.3389/fpubh.2026.1945221

**Published:** 2026-10-01

**Authors:** Yuhan Chen, Ning Wang, Anfa Yuan, Lei Xiang, Shiqi Long, Jianzhang Luo, Yang Liu

**Affiliations:** 1 School of Sports Science, Jishou University, Jishou, China; 2 Chengdu Vocational University of the Arts, Chengdu, China; 3 School of Physical Education, Xichang University, Xichang, China; 4 School of Physical Education and Arts, Hunan Medical College, Huaihua, China; 5 Chengdu Experimental Middle School, Chengdu, China

**Keywords:** childhood emotional maltreatment, executive function, network analysis, physical activity, social media addiction, university students

## Abstract

**Objective:**

To examine the association between childhood emotional maltreatment and social media addiction among university students, assess the indirect role of executive function and variation across levels of physical activity, and identify key features of the item-level network.

**Methods:**

Cross-sectional data were collected from 1,978 university students, who completed measures of childhood emotional maltreatment, executive function, social media addiction, and physical activity frequency over the previous 7 days. Models 4 and 8 of the SPSS PROCESS macro tested mediation and moderated mediation after adjustment for demographic covariates. Network analysis estimated item-level conditional associations and identified key nodes.

**Results:**

Childhood emotional maltreatment was positively associated with social media addiction. Executive function partially mediated this association, with an indirect effect of 0.173 accounting for 50.29% of the total effect. Interactions between childhood emotional maltreatment and physical activity were significant for executive function and social media addiction. At higher physical activity frequency, the positive direct association between childhood emotional maltreatment and social media addiction was weaker, whereas the negative association between childhood emotional maltreatment and executive function was stronger. In the item-level network, the strongest negative connection between childhood emotional maltreatment and executive function linked perceived hatred and emotional harm from family members to impulsive behavior without considering consequences. The strongest negative connection between executive function and social media addiction linked forgetting assigned tasks to functional impairment from excessive social media use. The emotional abuse item had the highest node strength and bridge strength, the working-memory item had the highest closeness centrality, and the social media addiction item reflecting an increasing urge to use social media had the highest one-step bridge expected influence.

**Conclusions:**

Childhood emotional maltreatment was associated with higher social media addiction, with poorer executive function statistically accounting for part of this association. Higher physical activity frequency was associated with a weaker positive direct association between childhood emotional maltreatment and social media addiction, but did not show a comparable buffering pattern for executive function. Emotional harm within the family, difficulties in inhibitory control and working memory, and an increasing urge to use social media were salient features of the item-level network.

## 1 Introduction

Child maltreatment remains a pervasive global concern, adversely affecting the developmental wellbeing of millions of children across both physical and psychological domains. The United Nations Convention on the Rights of the Child recognizes every child's right to protection from all forms of violence, including psychological violence (1). Among the various forms of maltreatment, emotional maltreatment is often particularly difficult to detect because it leaves no obvious physical injuries. Nevertheless, childhood emotional maltreatment (CEM) has been associated with persistent difficulties across multiple aspects of psychological development (2). CEM typically involves repeated non-physical behaviors by parents or primary caregivers, such as belittling, humiliation, rejection, intimidation, and emotional unresponsiveness. These behaviors convey the message that the child is worthless, unloved, unwanted, or valued only when meeting others' expectations, thereby undermining self-perception, emotion regulation, and social adjustment (3). Although emotional maltreatment is difficult to identify, its prevalence should not be underestimated. Previous research has shown that psychological maltreatment, including emotional abuse and emotional neglect, is among the most common forms of child maltreatment, with a global prevalence of up to 55% (4). A survey conducted in China also reported that 64.7% of university students had experienced physical or emotional maltreatment during childhood (5). Moreover, emotional maltreatment may have consequences that are at least as severe as, and in some cases more harmful than, those associated with other forms of abuse and neglect (6). Specifically, CEM is associated with a broad range of internalizing problems, including attachment disturbances, trauma-related symptoms, depression, anxiety, bulimic symptoms, and low self-esteem. It is also linked to externalizing and interpersonal difficulties, such as anger, aggression, relationship problems, involvement in violent relationships, substance misuse, and juvenile delinquency (7, 8). Given its hidden nature, relatively high prevalence, and persistent adverse effects on psychological and behavioral development, greater attention should be paid to CEM among university students.

Experiences of childhood maltreatment have been consistently associated with adverse emotional and behavioral outcomes. With the growing popularity of social media, these platforms have become deeply embedded in university students' academic activities, interpersonal interactions, and daily lives. They now serve as important channels for maintaining social connections, obtaining information, and expressing oneself. Social media addiction (SMA) refers to persistent, poorly controlled engagement with social media that is accompanied by psychological and physiological distress (9). Previous research has estimated the overall prevalence of SMA at approximately 24% (10). A meta-analysis covering 64 countries reported a pooled prevalence of 17.42% in the general population (11), while another study found that 15.2% of university students met the criteria for SMA (12). According to the 55th Statistical Report released by the China Internet Network Information Center (CNNIC), China has the largest social media user population worldwide (13), with university students accounting for a considerable share. Because university students routinely use social media for academic and everyday purposes, prolonged investment of time and attention may increase their risk of problematic social media use (14). Identifying the potential risk factors for SMA among university students is therefore essential. From the perspective of ecological systems theory (15), individual development is substantially shaped by the family environment, which constitutes a central microsystem. Within this framework, CEM represents an adverse family-context experience that may be associated with maladaptive behaviors, including SMA (16). According to the compensatory Internet use model, unmet psychological needs in offline settings, including needs for emotional support, self-worth, and social belonging, may be associated with a greater tendency to seek alternative gratification through online activities (17). Individuals with histories of maltreatment may therefore use social media to alleviate psychological distress and negative emotions. Such a coping pattern may be associated with greater psychological dependence on social media and a higher likelihood of addictive use. Research among university students has likewise linked physical or emotional maltreatment to Internet addiction (18). On this basis, H1 proposed that childhood emotional maltreatment would be positively related to SMA.

Executive function (EF) represents a theoretically relevant cognitive correlate of the association between CEM and SMA. EF refers to the capacity to coordinate multiple cognitive processes in support of goal-directed behavior and primarily comprises inhibitory control, working memory, and cognitive flexibility (19). During early development, the family constitutes the closest and most persistent microsystem, and the quality of parent–child relationships has been associated with the development of self-regulation and EF (20). Freud's trauma theory further proposes that childhood trauma may have enduring relevance across the life course, as traumatic memories may remain associated with later emotional, cognitive, and behavioral responses (21). Consistent with this theoretical perspective, CEM has been associated with poorer EF during adolescence and adulthood (22). The maturational imbalance model provides a theoretical explanation for the relevance of EF to SMA. During adolescence and early adulthood, the reward-related limbic system develops more rapidly, whereas the prefrontal cognitive-control system associated with EF matures more gradually (23, 24). This developmental imbalance may be associated with greater sensitivity to the immediate feedback and emotional rewards provided by social media. Accordingly, poorer inhibitory control and self-regulation may be associated with greater difficulty regulating social media use (25). Previous research has also identified EF as an important cognitive correlate of the association between childhood emotional maltreatment and later behavioral difficulties (26). Taken together, these perspectives suggest that EF is theoretically relevant to both CEM and SMA and may statistically account for part of their association. Accordingly, H2 proposed that CEM would be indirectly associated with SMA through EF.

Participation in physical activity (PA) at a moderate intensity contributes substantially to psychological functioning. PA encompasses bodily movements generated by skeletal muscle contraction that require energy expenditure, covering work-related movement, domestic chores, structured exercise, as well as recreational pursuits (27). Previous studies have shown that sustained participation in PA may attenuate the adverse effects of childhood adversity on corticolimbic circuits (28), enhance neuroplasticity (29), strengthen cognitive control (30) and consequently improve EF (31). According to self-efficacy theory (32), repeated denigration, verbal abuse, and emotional coldness associated with emotional maltreatment may gradually undermine children's self-esteem and contribute to learned helplessness. In contrast, PA provides repeated opportunities to overcome challenges and experience mastery, thereby strengthening self-confidence and self-efficacy (33). Physical activity may be associated with less severe psychological difficulties among individuals with histories of childhood emotional maltreatment, including better cognitive flexibility, self-regulation, and management of emotions and thoughts (34). Research among university students has also suggested that EF is a central component of psychological functioning and that moderate PA may partially alleviate impairments associated with childhood maltreatment (35). Within the cognitive-behavioral model of pathological Internet use, SMA is associated with the combined influence of distal and proximal factors (36). Distal factors include pre-existing psychopathology and situational cues, whereas proximal factors include maladaptive cognitions and negative affect. The model proposes that distal factors are associated with problematic Internet-related behaviors partly through proximal cognitions and negative affect. Within this framework, childhood maltreatment can be conceptualized as a distal situational factor associated with social media addiction alongside maladaptive cognitions and negative affect (37). Early traumatic experiences may be associated with distorted cognitive patterns and maladaptive beliefs, which can heighten psychological reliance on social media to address unmet emotional or relational needs. Such reliance may, in turn, be related to greater SMA. Based on this rationale, H3 proposed that the associations of childhood emotional maltreatment with executive function and social media addiction would differ across levels of physical activity.

Previous studies have primarily examined overall relationships among variables using scale-level total scores, which limits the ability to identify the roles of specific items. Network analysis can reveal conditional associations among individual items and identify central and bridge nodes. Therefore, the present study integrated conventional statistical methods with network analysis to examine the association between CEM and SMA at both the scale and item levels.

In summary, this study combined moderated mediation analysis with network analysis to examine the association between CEM and SMA among university students. The analysis examined whether EF mediated the association between CEM and SMA and whether PA moderated the associations of CEM with EF and SMA. The study further identified key intercommunity associations, central nodes, and bridge nodes, thereby providing an empirical basis for risk identification and targeted interventions for SMA among university students. Figure 1 illustrates the hypothesized model.

**Figure 1:**
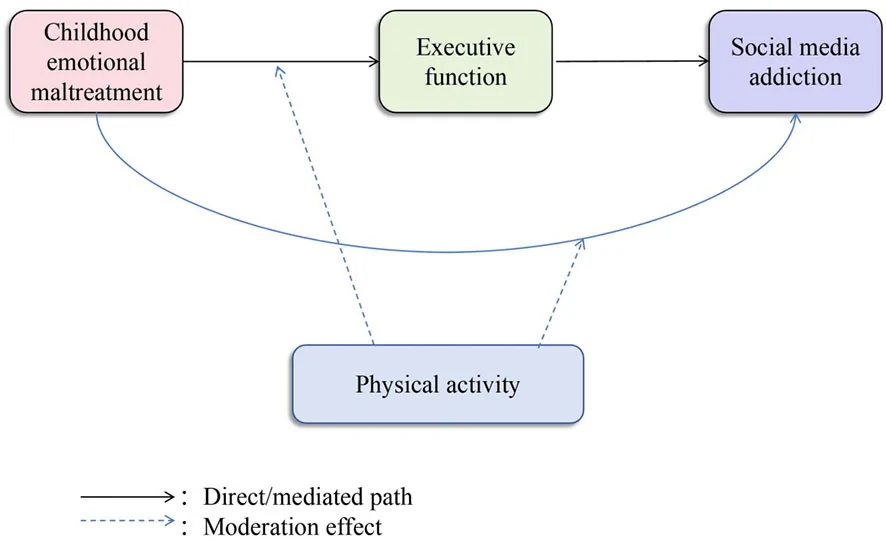
Hypothesized moderated mediation model.

## 2 Methods

### 2.1 Participants and procedure

In November 2025, participants were recruited using a combination of convenience-based and cluster-based sampling. Universities and classes across southwestern, northwestern, and central China were included according to accessibility through course instructors. Every student in the selected classes taught by the participating instructors was invited to take part in the survey, yielding 2,041 completed questionnaires. Two quality-control procedures were applied during data screening. First, questionnaires showing clear patterns of careless or invariant responding were identified based on response consistency, repetition, and unusually uniform response patterns across consecutive items. Particular attention was given to questionnaires in which identical or highly repetitive responses were selected across multiple items. Second, response quality was additionally evaluated using an attention-check question incorporated into the survey. Questionnaires from participants who failed this item were considered potentially unreliable and were excluded. These procedures were used to improve data quality and ensure that the retained responses adequately reflected the characteristics of the target population. Following data screening, 1,978 questionnaires were considered valid, yielding a valid questionnaire rate of 96.91%. Among the retained participants, 846 were men and 1,132 were women. Their average age was 18.98 years (SD = 1.13). Among the participants, 376 were only children and 1,602 had one or more siblings. Regarding paternal educational attainment, 107 participants reported an unknown level, 405 reported primary school education or below, 1,255 reported secondary school education, and 211 reported university education or above. For maternal educational attainment, the corresponding numbers were 123, 542, 1,122, and 191, respectively. Prior to survey administration, the research team informed participants about the study purpose, the material covered in the questionnaire, and how the collected information would be used. Participants were informed that the study involved no anticipated risks and that all collected information would remain strictly confidential. After receiving this information, participants voluntarily completed the questionnaire and provided written informed consent. The questionnaires were administered collectively within each participating class. Ethical approval for the study protocol was granted by the institutional review committee.

### 2.2 Measures

#### 2.2.1 Childhood emotional maltreatment

Childhood emotional maltreatment was assessed using a shortened four-item version of the Childhood Trauma Questionnaire Short Form (CTQ-SF), originally developed by Bernstein et al. (38) and adapted into Chinese by Zhao et al. (39). The measure covered emotional abuse and emotional neglect occurring before age 16, with two items assigned to each dimension. Responses to the emotional neglect items were reverse-coded. Responses were recorded on a five-point Likert scale from 1 (never) to 5 (always). Possible total scores ranged from 4 to 20, with higher values reflecting more severe childhood emotional maltreatment. Internal consistency was acceptable in the present sample (Cronbach's α = 0.701).

#### 2.2.2 Executive function

EF was assessed using the 10-item measure developed by Huang et al. (40), covering cognitive flexibility, inhibitory control, and working memory. Responses to each item were recorded on a three-point Likert scale, with scores ranging from 1 (often) to 3 (never). Higher total scores indicate better EF. In this study, the instrument demonstrated good internal consistency (Cronbach's α = 0.873).

#### 2.2.3 Social media addiction

SMA was measured using the Bergen SMA Scale (BSMAS), developed by Andreassen et al. (42) and later adapted for Chinese populations by Leung et al. (41). The six-item instrument uses a five-point Likert scale ranging from 1 (very rarely) to 5 (very often). Higher total scores indicate greater SMA severity. Luo et al. (43) proposed 24 as the optimal cutoff value. Participants scoring 24 or above were classified as screening positive for probable SMA, whereas those scoring below 24 were classified as being below the proposed cutoff. For the current sample, internal consistency was satisfactory, as indicated by a Cronbach's α of 0.843.

#### 2.2.4 Physical activity

PA was measured using a single item (44): “During the past 7 days, on how many days did you engage in at least 30 min of accumulated PA that increased your breathing rate?” Responses ranged from 0 to 7 days, with higher values indicating more frequent weekly PA. Given its short 7-day recall period and explicit 30-min activity criterion, this item was considered readily interpretable for university students and was selected to provide an efficient assessment of a clearly defined behavior in this large, multivariable survey. Brief measures, including single-item measures, may reduce completion time, cognitive burden, response fatigue, item non-response, and careless responding, and may be appropriate when the target construct or behavior is clearly defined (45–47). Accordingly, this variable was interpreted specifically as weekly PA frequency rather than as a comprehensive assessment of PA.

### 2.3 Data processing and analysis

Before the main analyses, multicollinearity diagnostics were conducted for CEM, EF, PA, and SMA to minimize potential bias arising from strong linear relationships among the study variables and to ensure reliable estimation of the path coefficients and interaction effects. Descriptive statistics and Pearson correlations were calculated in SPSS version 29.0, while potential common method bias was examined using Harman's single-factor test (48). A value below 40% for the variance explained by the initial unrotated factor indicated that substantial common method bias was unlikely. After controlling for gender, age, grade, paternal educational attainment, maternal educational attainment, and only-child status, mediation and moderated mediation models were estimated using the PROCESS macro for SPSS. Sampling region was not included as a covariate because Chinese universities generally enroll students from multiple provinces nationwide; therefore, the geographical location of the participating university does not reliably represent the region from which an individual student originates. Model 4 was used to examine the mediating role of EF, whereas Model 8 was used to test whether PA moderated the first-stage pathway of the mediation model and the direct association between CEM and SMA (49). All continuous variables were standardized before inclusion in the moderated mediation model. Bootstrap analysis based on 5,000 resamples was used to estimate 95% confidence intervals; statistical significance was defined as *p* < 0.05. Network analyses were conducted in *R* version 4.6.0 using the qgraph, bootnet, and networktools packages. The 21 nodes were assigned to four communities: childhood emotional maltreatment, EF, SMA, and PA. A regularized partial-correlation network was estimated using EBICglasso with cor_auto, pairwise complete observations, and an EBIC hyperparameter (γ) of 0.50. Following the methodological recommendation of Epskamp and Fried (50), γ = 0.50 was selected because this conservative setting prioritizes model parsimony and specificity, thereby limiting the retention of potentially spurious edges. Blue edges denoted positive conditional associations, whereas purple edges indicated negative associations; thicker edges reflected greater association strength. Node strength was treated as the primary centrality index. Closeness and betweenness were calculated as supplementary indices, and substantive interpretation was restricted to indices with correlation stability coefficients of at least 0.25. Bridge strength and one-step bridge expected influence were used to assess cross-community connectivity. Although IC, CF, and WM were visually distinguished using different colors, they were assigned to the same EF community in the bridge analysis. The stability of bridge strength and one-step bridge expected influence was evaluated using case-dropping bootstrap procedures with 2,000 resamples. Pairwise differences between nodes in these two bridge centrality indices were examined using non-parametric bootstrap difference tests with 2,000 resamples. Edge-weight accuracy was assessed using non-parametric bootstrapping, whereas the stability of ordinary node centrality indices was examined using case-dropping bootstrapping; both procedures involved 2,000 resamples. Correlation stability coefficients of 0.25 or above were regarded as acceptable, while those reaching 0.50 or higher reflected good stability.

## 3 Results

### 3.1 Multicollinearity test

Before conducting the moderated mediation analysis, multicollinearity diagnostics were performed for all study variables and the interaction term. The tolerance values for gender, age, grade level, parental educational attainment, only-child status, CEM, EF, PA, and the CEM × PA interaction ranged from 0.557 to 0.988. The VIF values for the corresponding variables ranged between 1.012 and 1.797. These values met the commonly accepted criteria for the absence of problematic multicollinearity (tolerance >0.10 and VIF <5). The collinearity diagnostics yielded a maximum condition index of 83.035, which exceeded the commonly used reference value. However, no single dimension showed high variance proportions for two or more variables simultaneously. This pattern indicates that the elevated condition index did not reflect severe multicollinearity. Overall, no substantial multicollinearity was detected among the variables, supporting their inclusion in the subsequent moderated mediation analysis (Table 1).

**Table 1:** Multicollinearity test.

Variable	Tolerance	VIF
Gender	0.915	1.092
Age	0.606	1.651
Father education	0.561	1.784
Mother education	0.557	1.797
Grade	0.603	1.658
Only child	0.955	1.048
CEM	0.877	1.141
EF	0.876	1.142
PA	0.940	1.064
CEM × PA	0.988	1.012

### 3.2 Common method bias test

Harman's single-factor test was conducted to evaluate the potential presence of common method bias. An unrotated exploratory factor analysis identified four components with eigenvalues above 1. The first unrotated factor accounted for 19.98% of the total variance, well below the recommended threshold of 40%. This result suggested that common method bias was unlikely to pose a substantial concern.

### 3.3 Descriptive analysis

Table 2 indicates significant gender differences in CEM, EF, SMA, and PA. Male students had higher scores for CEM, EF, and PA, whereas female students showed greater SMA. Across all four variables, no significant differences emerged between only children and students who had siblings. All four variables also differed significantly across grade levels. CEM and SMA scores were highest among senior students, while junior students showed the highest levels of EF and PA. In view of the differences observed across gender and grade level, all pre-specified demographic covariates were simultaneously included in the subsequent analyses, namely gender, age, grade level, paternal educational attainment, maternal educational attainment, and only-child status.

**Table 2:** Descriptive analysis of childhood emotional maltreatment, EF, SMA, and PA.

Variables		CEM		EF		SMA		PA	
		Mean	SD	Mean	SD	Mean	SD	Mean	SD
Gender	Male	8.48	3.29	22.80	4.14	14.56	4.94	3.59	2.00
	Female	8.20	3.00	22.08	3.55	15.12	4.39	3.00	1.87
	t	1.97^*^		4.02^***^		−2.62^**^		6.67^***^	
Only child status	Only child	8.33	3.29	22.34	3.90	14.89	4.55	3.32	1.99
	Non-only child	8.32	3.09	22.40	3.81	14.87	4.66	3.23	1.94
	t	0.07		−0.30		0.08		0.84	
Grade	Freshman	7.98	2.89	22.31	3.67	14.39	4.50	3.56	1.95
	Sophomore year	8.69	3.23	22.29	3.91	15.37	4.69	2.83	1.83
	Junior	7.64	3.58	23.86	4.22	14.25	4.68	4.63	1.94
	Senior	9.86	4.22	23.00	3.37	16.14	7.84	1.57	1.62
	F	10.66^***^		5.69^***^		7.83^***^		45.02^***^	

^*^ <0.05; ^**^ <0.01; ^***^ <0.001.

### 3.4 Correlation analysis

As shown in Table 3, CEM was negatively correlated with EF and PA but positively correlated with SMA. EF showed a negative correlation with SMA and a positive correlation with PA, whereas SMA was negatively correlated with PA. All observed correlations were statistically significant.

**Table 3:** Correlation analysis.

Variables	M ±SD	1	2	3	4
CEM	8.32 ± 3.13	1			
EF	22.39 ± 3.83	−0.310^***^	1		
SMA	14.88 ± 4.64	0.253^***^	−0.435^***^	1	
PA	3.25 ± 1.95	−0.138^***^	0.136^***^	−0.172^***^	1

^***^ <0.001.

### 3.5 Mediation model test

After adjustment for demographic covariates, Table 4 and Figure 2 showed a positive association between childhood emotional maltreatment and social media addiction. After executive function was included in the model, the direct association between childhood emotional maltreatment and social media addiction remained statistically significant but was reduced in magnitude. Childhood emotional maltreatment was negatively associated with executive function, which was also negatively associated with social media addiction. Bootstrap analysis indicated a significant indirect association through executive function, accounting for approximately half of the total association (Table 5).

**Table 4:** Mediation model test.

Outcome variables	Predictor variables	β	SE	*t*	*R^2^*	*F*
SMA	CEM	0.344	0.033	10.594^***^	0.090	24.473^***^
EF	CEM	−0.369	0.026	−14.031^***^	0.122	34.181^***^
SMA	CEM	0.171	0.032	5.429^***^	0.221	62.182^***^
	EF	−0.468	0.026	−18.194^***^	—	—

^***^ <0.001.

**Figure 2:**
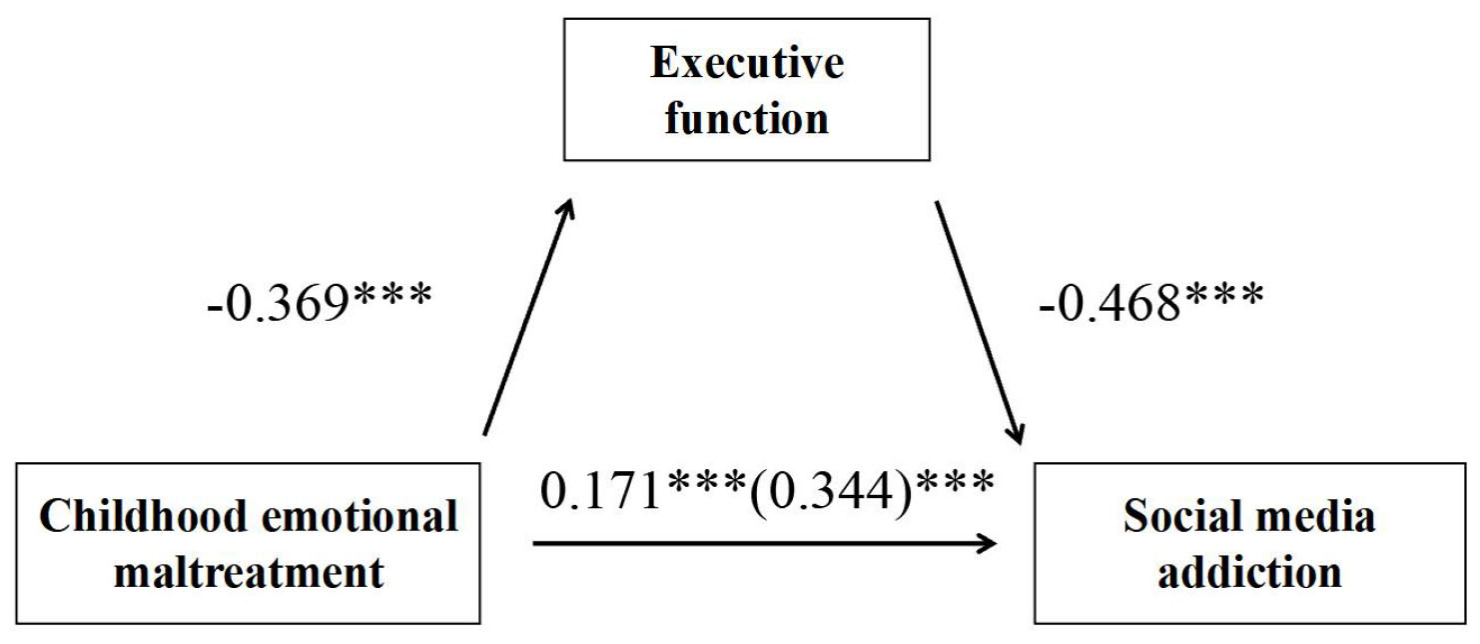
Mediation model diagram. *** <0.001

**Table 5:** Mediation model path analysis.

Intermediate path	Effect size	SE	Bootstrap 95% CI	Proportion of mediating effect
Total effect	0.344	0.033	0.280, 0.408	—
Direct effect	0.171	0.032	0.109, 0.233	49.71%
Indirect effect	0.173	0.017	0.140, 0.207	50.29%

### 3.6 Moderated Mediation Test

As presented in Tables 6, 7 and Figure 3, CEM was negatively associated with EF but positively associated with SMA, while EF was negatively associated with SMA. Significant interaction terms between childhood emotional maltreatment and physical activity were observed for both executive function and social media addiction, indicating that these associations differed across levels of physical activity. Simple slope analysis showed that the negative association between CEM and EF remained significant at low, moderate, and high PA levels and became stronger as PA increased. In contrast, CEM remained positively associated with SMA across all three PA levels, although the strength of this association gradually declined as PA rose. These findings indicate that PA weakened the direct association between CEM and SMA but did not exhibit a similar buffering pattern for the association between CEM and EF.

**Table 6:** Moderated mediation model test.

Variables	EF			SMA		
	β	SE	t	β	SE	t
CEM(A)	−0.300	0.022	−13.937^***^	0.116	0.021	5.480^***^
PA (B)	0.081	0.022	3.749^***^	−0.096	0.021	−4.664^***^
CEM × PA	−0.048	0.021	−2.322^*^	−0.039	0.020	−1.976^*^
EF				−0.388	0.021	−18.286^***^
*R* ^2^	0.124			0.223		
F	31.050^***^			56.437^***^		

^*^ <0.05, ^***^ <0.001.

**Table 7:** Simple slope analyses of PA's moderating effects.

Moderated path	PA levels	Effect size	SE	*t*	Lower limit 95% CI	Upper limit 95% CI
PA moderates the association between CEM and EF						
CEM → EF	Low	−0.251	0.031	−8.223^***^	−0.311	−0.191
CEM → EF	Medium	−0.300	0.022	−13.937^***^	−0.342	−0.257
CEM → EF	High	−0.348	0.029	−11.880^***^	−0.405	−0.291
PA moderates the association between CEM and SMA						
CEM → SMA	Low	0.155	0.029	5.300^***^	0.098	0.213
CEM → SMA	Medium	0.116	0.021	5.480^***^	0.075	0.158
CEM → SMA	High	0.078	0.029	2.714^**^	0.022	0.134

^**^ <0.01, ^***^ <0.001.

**Figure 3:**
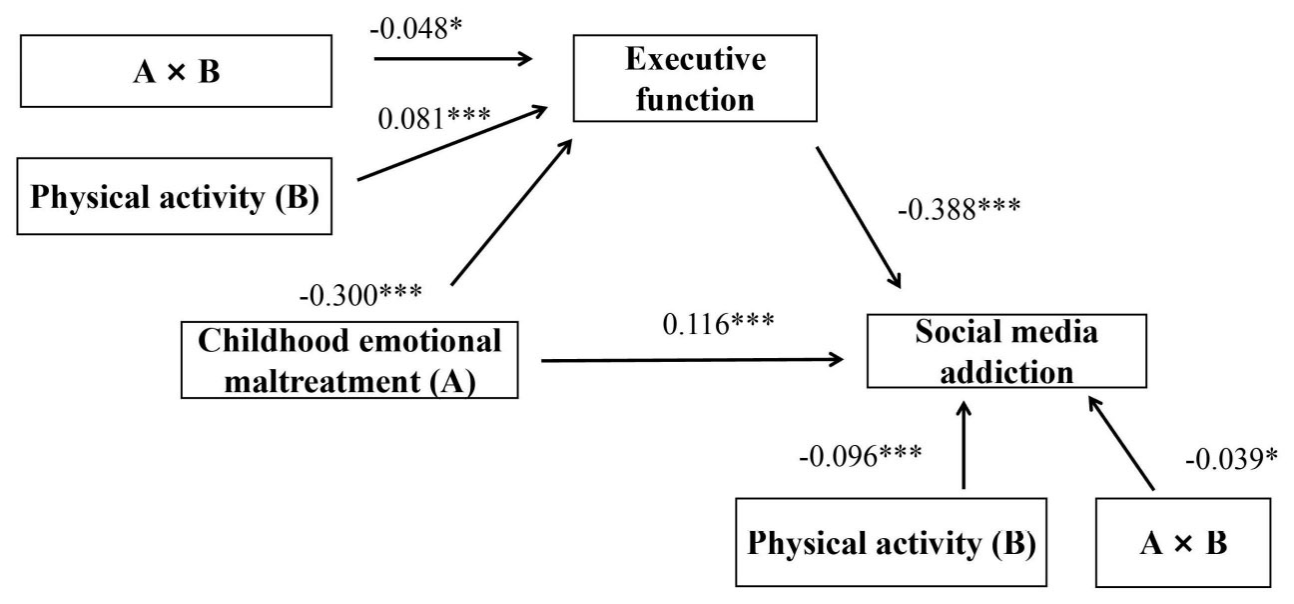
Moderated mediation model (* <0.05, *** <0.001).

### 3.7 Johnson–Neyman analysis

The Johnson–Neyman method identified the PA values at which the direct association of CEM with SMA became statistically significant. As shown in Figure 4, no significance threshold was identified for the CEM–EF pathway, indicating that this association remained statistically significant across all observed levels of PA. By contrast, a critical standardized PA value of 1.3228 was identified for the CEM–SMA pathway. Based on the sample mean and standard deviation of PA (M = 3.25, SD = 1.95), this value corresponded to approximately 5.83 physically active days per week (3.25 + 1.3228 × 1.95). Because responses were recorded as whole numbers from 0 to 7 days, this threshold can be interpreted as approximately 6 days per week. The direct association between CEM and SMA was statistically significant below this threshold but was non-significant above it.

**Figure 4:**
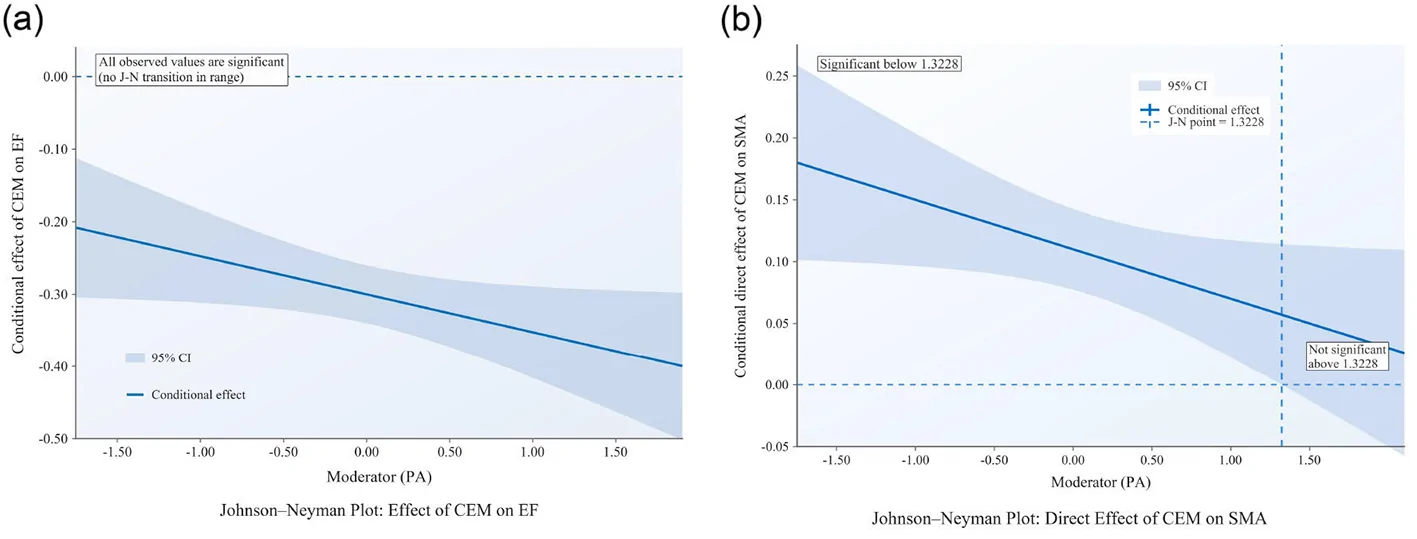
Johnson–Neyman moderation effect analysis. **(a)** Conditional relation of childhood emotional maltreatment to EF. **(b)** Conditional direct relation of childhood emotional maltreatment to SMA. Shaded bands indicate 95% confidence intervals.

### 3.8 Network analysis of the main variables

The network analysis results (Figure 5) showed that, among the cross-community associations, the strongest connection between CEM and EF was the negative association between IC3 and EA2 (*r*_*p*_ = −0.119). The strongest association between EF and SMA was the negative connection between WM3 and SMA6 (*r*_*p*_ = −0.084). PA was directly connected to 11 nodes. Its strongest positive association was with IC2 (*r*_*p*_ = 0.051), whereas relatively strong negative associations were observed with EN2 (*r*_*p*_ = −0.044) and SMA5 (*r*_*p*_ = −0.041).

**Figure 5:**
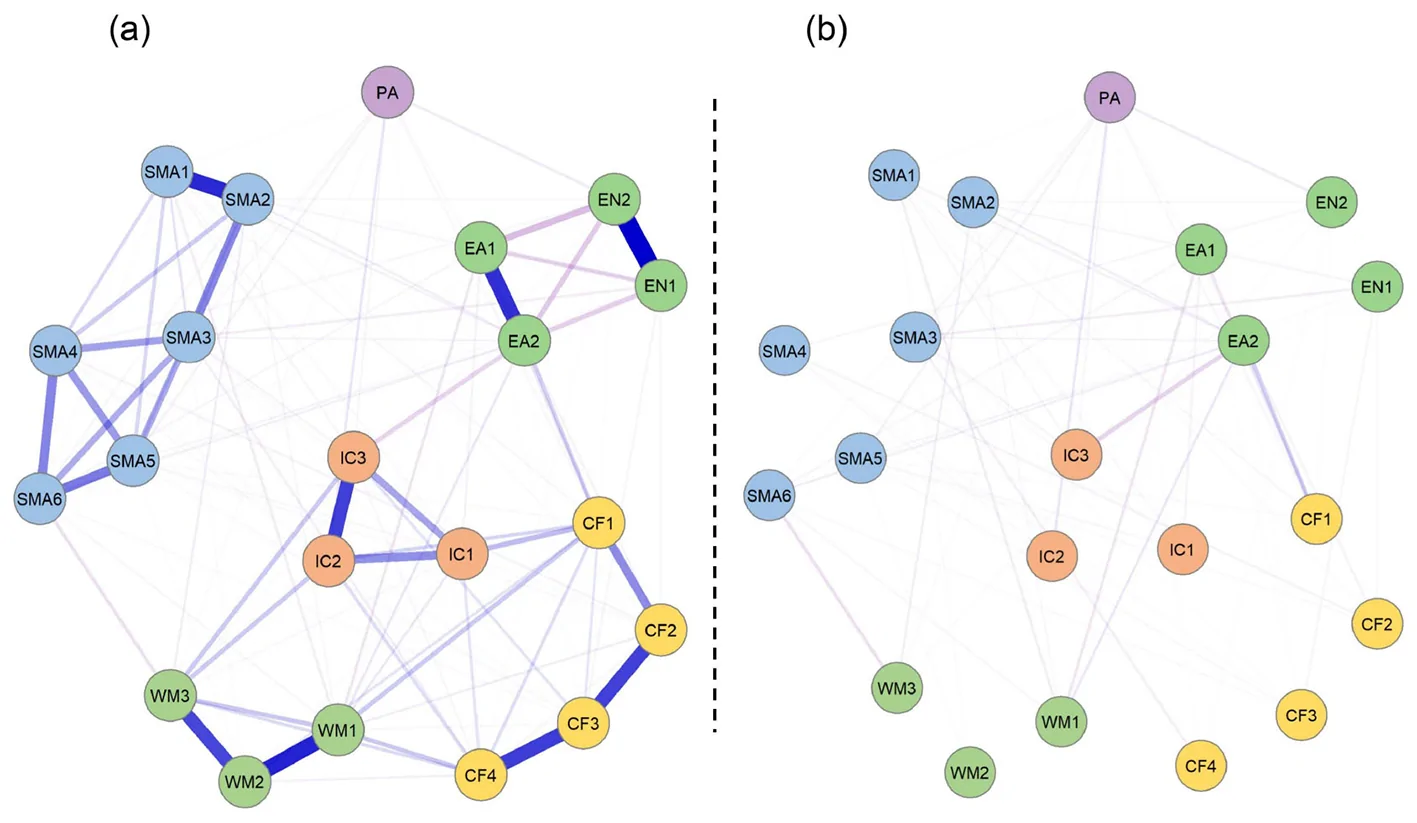
Cross-sectional network analysis. **(a)** Regularized partial correlation network; **(b)** cross-community network. Nodes represent individual items, and node colors distinguish the major variable domains and subdomains. Blue edges denote positive conditional associations, while purple edges represent negative conditional associations. Greater edge thickness reflects a stronger association. EA, emotional abuse; EN, emotional neglect; IC, inhibitory control; CF, cognitive flexibility; WM, working memory; SMA, social media addiction; PA, physical activity.

Bridge centrality analysis (Figure 6) showed that the emotional abuse item “I felt that someone in my family hated me and caused me emotional pain or trauma” (EA2) had the highest bridge strength (raw estimate = 0.488). The social media addiction item “I felt an urge to use social media more and more” (SMA2) had the highest estimated one-step bridge expected influence (raw estimate = 0.067). Case-dropping bootstrap analyses yielded CS coefficients of 0.361 for bridge strength and 0.439 for one-step bridge expected influence, indicating acceptable stability. Non-parametric bootstrap difference tests showed that the bridge strength of EA2 was significantly higher than that of all other nodes included in the pairwise comparisons. SMA2 had significantly greater one-step bridge expected influence than 12 other nodes; however, its differences from CF1, EA2, EN1, IC2, SMA6, WM2, and WM3 were not significant. Thus, EA2 showed the greatest absolute cross-community connectivity, whereas SMA2 showed the largest estimated positive net cross-community connectivity.

**Figure 6:**
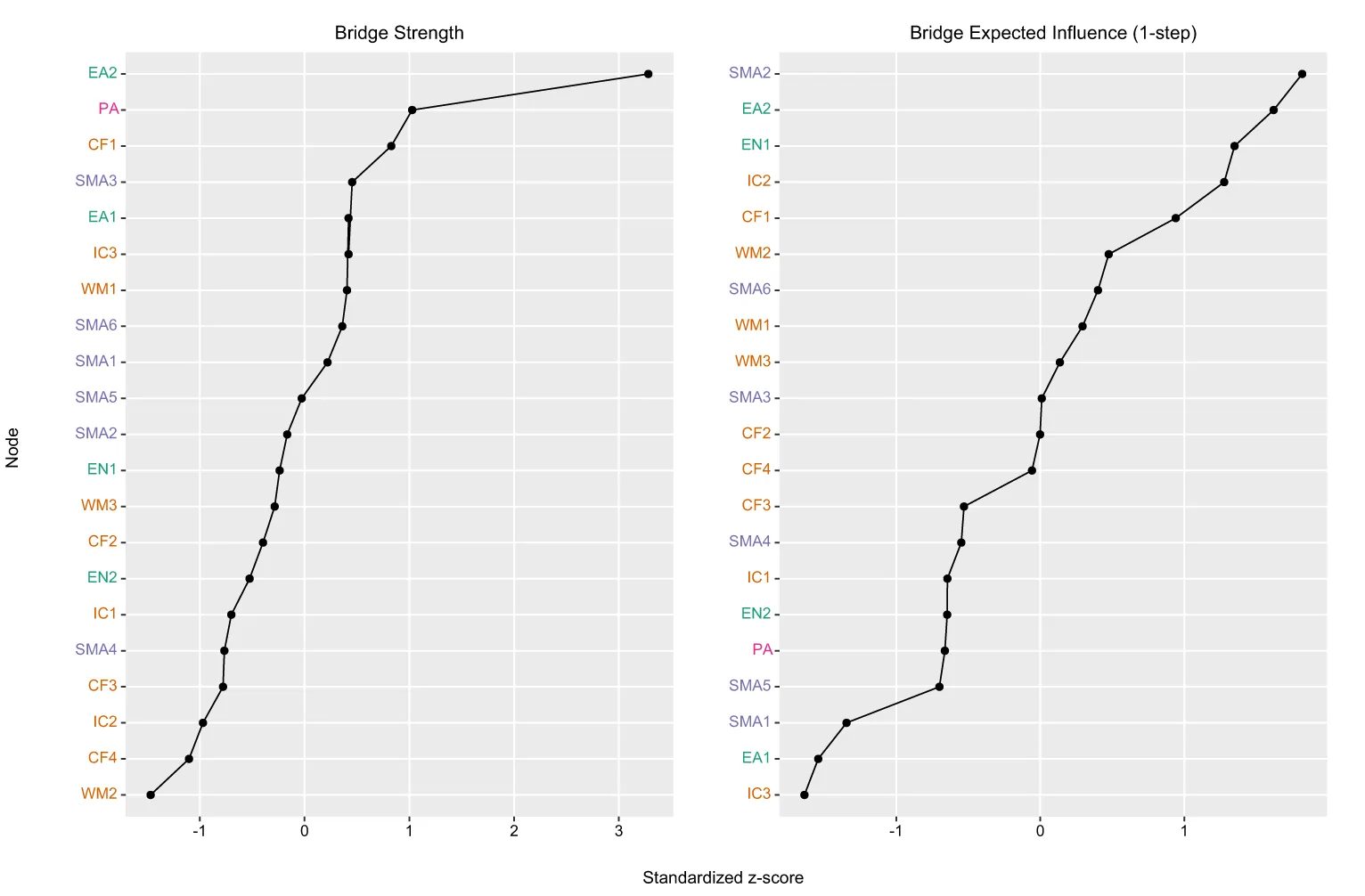
Bridge centrality estimates. The left panel presents standardized bridge strength, and the right panel presents standardized one-step bridge expected influence. Bridge strength represents the sum of the absolute cross-community edge weights, whereas bridge expected influence retains the direction of the cross-community associations.

The node centrality estimates (Figure 7) showed that EA2 had the highest strength, indicating the strongest overall connectivity with other nodes. WM3 had the highest closeness estimate, although this result should be interpreted cautiously in light of the stability analysis. Betweenness estimates are presented in Figure 7 for completeness but were not substantively interpreted. Given the good stability of node strength, EA2 may be regarded as a comparatively stable high-strength node in the network.

**Figure 7:**
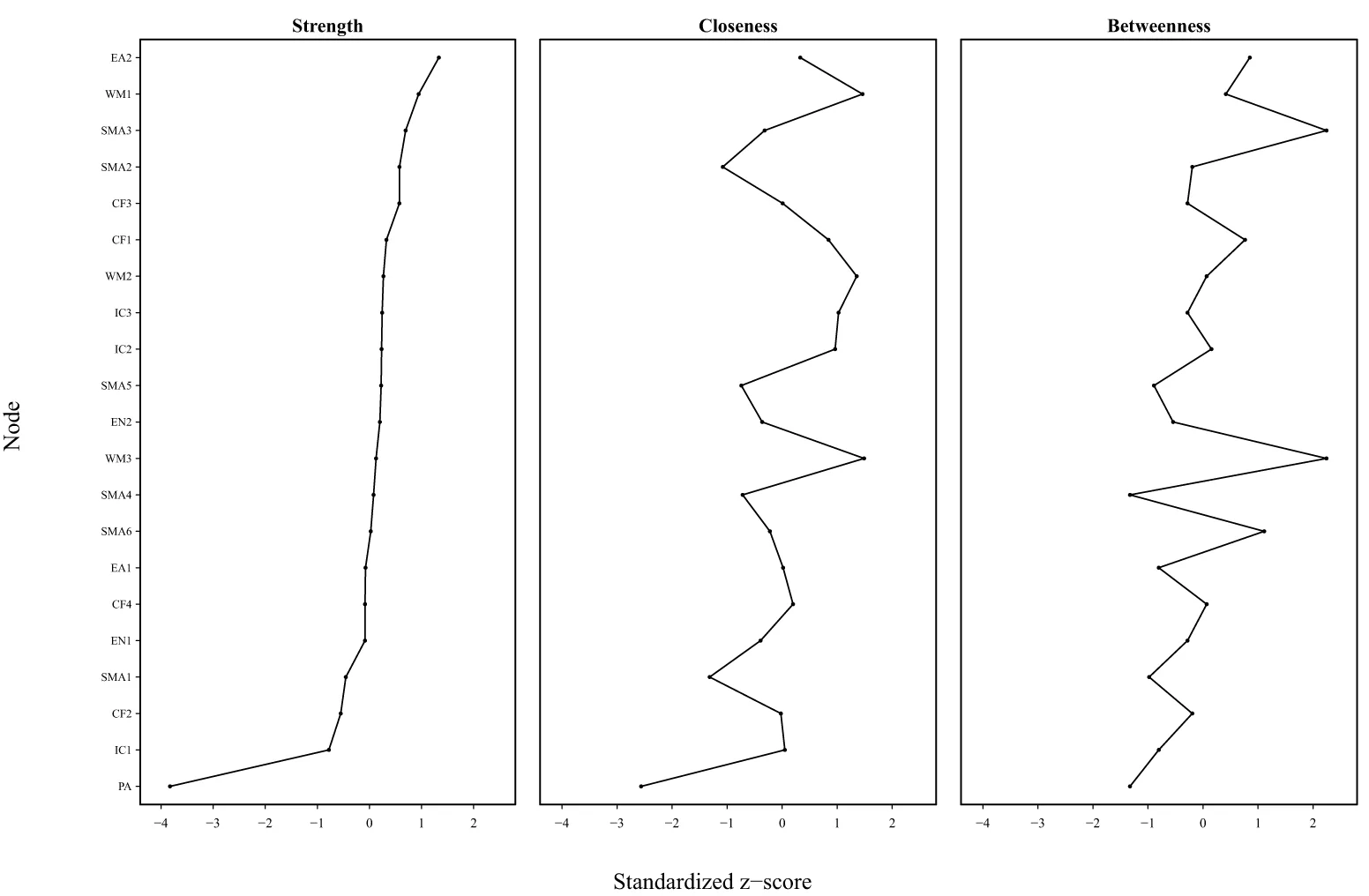
Centralized measurement of network nodes. Centrality indices are presented as standardized *z*-scores.

The stability analysis (Figure 8) indicated good stability for node strength (CS = 0.750) and acceptable stability for closeness centrality (CS = 0.439). In contrast, betweenness centrality showed poor stability (CS = 0.128); consequently, no substantive conclusions were drawn from this index.

**Figure 8:**
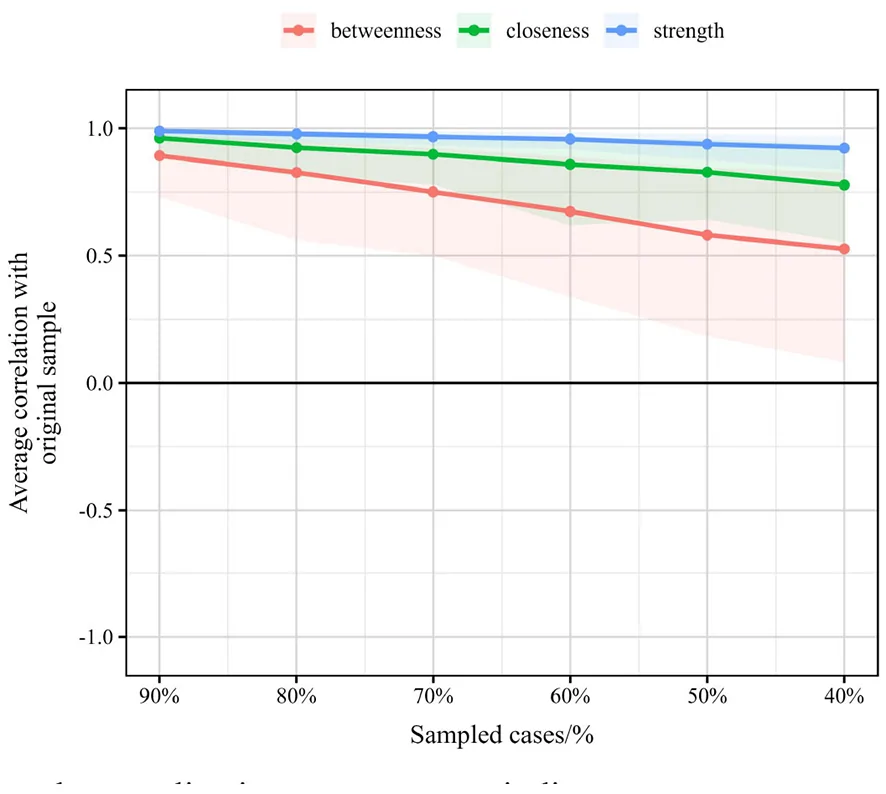
Stability testing of network centralization measurement indicators.

## 4 Discussion

This study combined a moderated mediation model with network analysis to examine the relationships among CEM, EF, PA, and SMA among university students at both the scale and item levels. The results showed that CEM was positively associated with SMA, with EF serving as a partial mediator. Higher levels of physical activity were associated with better executive function, and the positive association between childhood emotional maltreatment and social media addiction was weaker at higher levels of physical activity. Network analysis further highlighted emotional harm within the family, difficulties in inhibiting poorly considered behavior, and working-memory problems as prominent features of the network. EA2 showed the strongest absolute cross-community connectivity, whereas SMA2 showed the highest positive net cross-community connectivity.

### 4.1 Positive association between childhood emotional maltreatment and SMA among university students

CEM was positively associated with SMA, while the direct effect accounted for 49.71% of the total effect. Thus, H1 was supported, consistent with previous findings (51). This result can be interpreted through attachment theory. Individuals exposed to emotional maltreatment during childhood often lack stable caregiving support and secure emotional bonds, which may exert lasting adverse effects on their cognitive functioning, emotion regulation, and behavioral patterns (52). When adequate emotional support is unavailable in their offline lives, these individuals may be more likely to use social media to seek emotional comfort, social support, and validation of self-worth. Repeated reliance on social media for these purposes may gradually strengthen psychological dependence and increase the risk of addictive use. Early childhood environments may also shape the development of life-history strategies. Unsupportive or unpredictable environments may promote a faster life-history strategy (53), characterized by a stronger preference for immediate rewards and short-term behavioral orientations (54, 55). Social media use may reflect this reward-oriented tendency because social media platforms offer convenient access, immediate feedback, and highly engaging content. These features can provide rapid emotional compensation and short-term gratification, making repeated use particularly appealing to individuals with a faster life-history orientation. Through continued reinforcement, such patterns may develop into addictive behavior (56). Prior research has linked CEM to traditional mental and physical health concerns, including anxiety, insomnia, depression, and suicidal behavior (57, 58), as well as newer behavioral problems such as SMA (59, 60). Collectively, this evidence identifies CEM as an important early adverse experience associated with SMA among university students. The network analysis showed that the item “I felt that someone in my family hated me and caused me emotional pain or trauma” had the highest node strength and bridge strength, indicating the strongest overall connectivity and absolute cross-community connectivity in the network. The emotional abuse experience represented by this item may therefore occupy a prominent position both within the overall network and in linking different variable communities.

### 4.2 The mediating role of executive function in the association between childhood emotional maltreatment and SMA among university students

This study found that EF significantly mediated the association between CEM and SMA among university students. The indirect association represented 50.29% of the total association, indicating that EF may partly account for the relationship between childhood emotional maltreatment and SMA. This finding supports H2. From a neurobiological perspective, childhood maltreatment has been associated with alterations in brain regions and neural pathways involved in processing and transmitting aversive experiences. It has also been associated with structural differences in the anterior cingulate cortex, dorsolateral prefrontal cortex, orbitofrontal cortex, corpus callosum, and hippocampus (61). The prefrontal cortex supports attentional control, inhibitory control, working memory, and cognitive flexibility. Childhood emotional maltreatment has been associated with differences in prefrontal–limbic circuitry and related neural networks, as well as with difficulties in attentional control, impulse inhibition, emotion regulation, and goal-directed behavior. These difficulties may manifest as poorer EF. Meanwhile, social media platforms provide frequent and immediate rewards through likes, comments, message notifications, personalized recommendations, and continuously updated content. Individuals with poorer EF may have greater difficulty suppressing urges to use social media and may therefore be more vulnerable to excessive or compulsive use. The network analysis further clarified the item-level associations underlying this indirect pathway. The item “I felt that someone in my family hated me and caused me emotional pain or trauma” showed a strong negative association with “I behaved excessively or acted without considering the consequences.” Exposure to childhood environments characterized by rejection, neglect, or threat has been associated with heightened sensitivity to stress and negative affect and with greater difficulty regulating emotions and behavior under conditions of emotional arousal. According to stress sensitization theory, early adversity heightens sensitivity to subsequent stressors and increases vulnerability to emotion-regulation difficulties in response to negative experiences (62). Because inhibitory control enables individuals to resist distractions, suppress inappropriate responses, and maintain goal-directed behavior, those exposed to greater emotional maltreatment during childhood may experience greater difficulty regulating their behavior under stress. This may help explain the strong association between EA2 and IC3.

In addition, “I often forgot tasks or responsibilities assigned by others” (WM3) showed a relatively strong negative association with “My excessive use of social media negatively affected my study or work” (SMA6). Although WM3 had the highest closeness estimate, this finding should be interpreted cautiously because the stability of closeness was only acceptable, whereas the unstable betweenness estimates were not interpreted. Therefore, the WM3–SMA6 connection is treated as an item-level association requiring replication rather than evidence that WM3 is a central node in the network. These network findings were consistent with the regression analysis and showed that childhood emotional maltreatment, social media addiction, and difficulties in executive-function components, particularly inhibitory control and working memory, were interconnected. Bridge centrality analysis further showed that EA2 had the highest bridge strength, reinforcing the prominent cross-community position of emotional harm within the family. In contrast, SMA2, reflecting an increasing urge to use social media, had the highest one-step bridge expected influence. Because bridge strength is based on the absolute values of cross-community edges, whereas bridge expected influence retains their signs, these results indicate that EA2 had the greatest total absolute cross-community connectivity and SMA2 had the largest positive net cross-community connectivity. This pattern highlights emotional harm within the family and an increasing urge to use social media as salient features connecting the variable communities. Given the cross-sectional design, these associations should not be interpreted causally.

### 4.3 Physical activity as a moderator of the association between childhood emotional maltreatment and executive function

The association between CEM and EF differed according to PA levels. However, the observed pattern did not reflect the expected straightforward protective effect and was not fully consistent with some previous findings. Several explanations may account for this unexpected pattern. First, the psychological benefits associated with PA are not uniform but may vary according to activity domain, dose, participation context, and motivation. Previous research has shown that the association between PA and mental health differs across activity domains and may not follow a simple linear dose–response pattern, while autonomous motivation is particularly important for sustained and beneficial participation (63–65). In the university context, the same weekly PA frequency may therefore represent qualitatively different experiences, ranging from self-directed and enjoyable leisure-time activity to participation driven by physical education requirements, social expectations, or attempts to cope with current distress. One possible explanation is compensatory self-selection: students experiencing greater trauma-related distress or EF difficulties may participate in PA more frequently as a coping strategy or in response to external requirements. Because the present data are cross-sectional, this possibility cannot be distinguished from a genuine moderating process. Accordingly, the result does not indicate that PA is harmful.

Second, the PA item assessed only the number of days on which participants accumulated at least 30 min of activity that increased their breathing rate. It did not distinguish activity type, exact intensity and duration, degree of structure, participation motivation, or subjective experience. Consequently, students engaging in substantially different forms of PA could receive the same weekly-frequency score. This limited differentiation may have obscured the potentially protective characteristics of PA and may partly account for the unexpected interaction. Nevertheless, the present study cannot determine the extent to which the finding reflects measurement characteristics, compensatory participation, residual confounding, or bidirectional associations. The item-level association between PA and inhibitory control in the network analysis does not contradict the moderation result because the former represents a partial association between specific items, whereas the latter concerns variation in the association between CEM and overall EF across levels of weekly PA frequency. Therefore, this finding should be regarded as preliminary and hypothesis-generating. Future longitudinal and experimental studies should combine multidimensional PA questionnaires with objective measures, such as accelerometers or activity records, and distinguish activity type, intensity, duration, context, motivation, and affective experience.

### 4.4 Physical activity as a moderator of the association between childhood emotional maltreatment and SMA

The findings indicated that PA moderated the association between CEM and SMA among university students, consistent with prior research (66). SMA is a form of problematic Internet-related behavior characterized by excessive use, impaired control, and functional disruption. It can adversely affect university students' academic functioning, emotion regulation, and social adjustment. CEM is widely regarded as an important early adverse experience associated with an increased risk of SMA (67). The findings indicate that PA may attenuate this association through several physiological processes. Regular participation in PA can support hypothalamic–pituitary–adrenal axis regulation, limit excessive stress responses, and stimulate the release of endorphins, dopamine, and serotonin (68). Endorphins may improve mood and alleviate negative emotional states associated with CEM, thereby reducing the need to rely excessively on social media for emotional comfort (69). Dopamine and serotonin play important roles in reward processing, mood stabilization, and motivational regulation. Their regulation through PA may enhance positive emotional experiences and reduce the tendency to seek immediate rewards through social media (70). PA may also increase brain-derived neurotrophic factor (BDNF) production, promote neuroplasticity, and strengthen prefrontal cognitive control, potentially improving the regulation of impulsive social media use (71). Psychosocial mechanisms may also contribute to this buffering effect. Through mastery experiences and improvements in physical competence, PA can enhance self-efficacy and self-identity while providing university students with histories of emotional maltreatment with healthier outlets for emotional expression and additional sources of social support (72). Activities such as running, ball games, and fitness training may redirect attention away from distressing experiences. Goal attainment and peer interaction during exercise may also strengthen individuals' sense of control and belonging in offline life, thereby reducing compensatory dependence on social media (73). PA may therefore weaken the adverse association between CEM and SMA through multiple pathways, including physiological regulation, emotional improvement, enhanced cognitive control, and greater social support. These findings provide further support for H3.

The Johnson–Neyman analysis further indicated that the direct association between CEM and SMA differed across levels of PA. The association was no longer statistically significant when the standardized PA score reached 1.3228, corresponding to approximately 5.83 physically active days per week, or approximately 6 days on the original integer scale. Descriptively, the direct association remained significant among students reporting 0–5 physically active days per week but was non-significant among those reporting 6–7 days. However, this model-derived region of statistical significance should not be interpreted as establishing a causal protective threshold, a clinical cutoff, or a minimum effective dose of PA. It represents a conditional association observed in the present cross-sectional sample. Longitudinal and intervention studies using multidimensional and device-based PA assessments are needed to determine whether this pattern can be replicated and whether a meaningful activity threshold exists.

### 4.5 Strengths and implications

This study integrated moderated mediation analysis with network analysis. At the scale level, it examined the roles of EF and PA, while at the item level, it identified specific cross-community associations, central nodes, and bridge nodes. This combined approach addressed the limitations of relying solely on total scores, which may obscure heterogeneity within multidimensional constructs. The findings provide more specific guidance for identifying university students who have experienced emotional harm within the family and who exhibit difficulties in inhibiting behavior, considering behavioral consequences, or maintaining information in working memory. Theoretically, future research should further examine the distinct roles of different executive-function components. EF comprises inhibitory control, working memory, and cognitive flexibility, and these components may differ in the extent to which they mediate the association between CEM and SMA. Future studies should also adopt more comprehensive measures of PA by distinguishing aerobic exercise, resistance training, team sports, and individual activities, as well as exercise intensity, frequency, duration, motivation, and subjective experience. Such refinements may clarify why PA exhibits different moderating patterns across specific pathways. In practice, neither university students with a history of CEM nor university educators can alter past experiences. Nevertheless, the plasticity of EF provides an important focus for supporting positive changes in current cognitive and behavioral functioning. Existing intervention evidence offers preliminary guidance for incorporating PA into university-based programs. A systematic review and meta-analysis of 19 randomized comparisons indicated that exercise interventions were associated with lower levels of Internet addiction, anxiety, loneliness, stress, and other psychological symptoms among college students. Open-skill activities and programs combining open- and closed-skill activities showed relatively favorable outcomes, although substantial heterogeneity was observed across studies (74). In one randomized trial, an 8-week program of moderate-intensity brisk walking or cognitively engaging exercise, delivered for 50 min twice weekly, was associated with improvements in inhibitory control and general frontal functioning, although changes in working memory and cognitive flexibility were non-significant (75). Another randomized trial found that basketball or Baduanjin, delivered for 90 min three times weekly over 12 weeks, was associated with reductions in problematic smartphone use and several related psychological symptoms among Chinese college students, with most improvements maintained at the 2-month follow-up (76). Taken together, these findings provide an empirical basis for universities to evaluate supervised and progressive programs involving aerobic exercise, team sports, or mind–body exercise, with specific formats adapted to students' health status and exercise preferences. Potential pathways may include better inhibitory control, lower craving and negative affect, greater mastery and social connection, and the replacement of unstructured screen time with goal-directed activity. Within such programs, screening and support may pay particular attention to students reporting emotional harm within the family or an increasing urge to use social media, as these items showed the highest bridge strength and one-step bridge expected influence, respectively.

These practical implications should be interpreted cautiously. Internet addiction and problematic smartphone use are related to, but not equivalent to, SMA, and the present cross-sectional study using a single-item measure of weekly PA frequency cannot determine an optimal exercise type or dose. Therefore, the intervention parameters reported above should be regarded as evidence-informed examples rather than specific exercise prescriptions. Future randomized trials should directly compare exercise type, intensity, frequency, and duration and examine EF and emotion regulation as potential mediators of intervention-related changes in SMA. The present moderated mediation framework may also be extended by incorporating early maladaptive schemas and body-awareness processes. Examining early maladaptive schemas, EF, emotion regulation, PA, and SMA within a unified model may provide a broader understanding of their interrelationships (77, 78). Intervention studies could further evaluate multicomponent programs combining schema-focused strategies, EF and emotion-regulation training, body-awareness practices, and appropriately structured PA.

### 4.6 Limitations

Despite its theoretical and practical contributions, several limitations should be acknowledged. CEM was measured using retrospective self-reports, which may have been affected by recall bias or participants' emotional states at the time of assessment. Future studies should use longitudinal designs and incorporate reports from parents, caregivers, or other knowledgeable informants to improve the accuracy of assessments of adverse childhood experiences. Second, CEM was assessed using a brief four-item measure. Although the measure demonstrated acceptable internal consistency and reduced participant burden, its limited number of items may constrain the breadth and precision of the assessment. Future studies should replicate the findings using more comprehensive CEM measures. Third, PA was assessed using a validated single item that efficiently captured weekly activity frequency but did not provide detailed information on activity type, intensity, total duration, regularity, or participation motives. Future studies should combine multidimensional PA questionnaires with accelerometers, wearable devices, and exercise logs. Longitudinal networks, cross-lagged network models, and experimental designs are also needed to verify the findings. In addition, although the participating universities were located in southwestern, northwestern, and central China, Chinese universities generally enroll students from multiple provinces nationwide. Consequently, university location was not treated as a participant-level indicator of regional background. Future studies should collect information on the regions from which students originate and directly examine potential regional differences. Finally, the cross-sectional design and convenience sampling precluded conclusions about temporal ordering or causality and limited the generalizability of the findings to other cultural contexts and age groups. Future studies should adopt longitudinal, cross-lagged, or experimental designs and examine the proposed moderated mediation model in larger, more diverse, and representative samples. Addressing these limitations will deepen understanding of the processes associated with SMA among university students with a history of CEM.

## 5 Conclusions

Childhood emotional maltreatment was associated with higher levels of SMA among university students, and a significant indirect association through EF was observed. The direct association between childhood emotional maltreatment and social media addiction was weaker at higher levels of physical activity, whereas the negative association between childhood emotional maltreatment and executive function was stronger. At the item level, the item reflecting feelings of being hated or emotionally hurt by a family member showed the highest node strength and bridge strength. Working-memory difficulties were closely associated with functional impairment related to excessive social media use, whereas the item reflecting an increasing urge to use social media showed the highest estimated one-step bridge expected influence. Taken together, these findings provide a more differentiated understanding of the relationships among childhood emotional maltreatment, EF, PA, and SMA at both the construct and item levels, and may contribute to more precise risk identification and support planning for university students with histories of childhood emotional maltreatment.

## Data Availability

The raw data supporting the conclusions of this article will be made available by the authors, without undue reservation.
